# Data Analytics Dashboard for Pre–Magnetic Resonance Imaging Safety Screening of Implantable Medical Devices

**DOI:** 10.2196/90391

**Published:** 2026-09-23

**Authors:** Ee Chin Teo, Hsin-Yu Jen, Eric Fang, Yonghan Ting, Andrew Makmur, James Hallinan

**Affiliations:** 1 Department of Diagnostic Imaging, National University Hospital, 5 Lower Kent Ridge Road, Singapore, 119074, Singapore, 65 67722535; 2 Innovation Office, Academic Informatics Office, National University Health System, Singapore, Singapore; 3 Department of Diagnostic Radiology, Yong Loo Lin School of Medicine, National University of Singapore, Singapore, Singapore

**Keywords:** magnetic resonance imaging, MRI, MRI safety, safety screening, implantable medical device, pacemaker, stimulator, dashboard, data analytics, analytics

## Abstract

This research letter summarizes the development and deployment of a data analytics dashboard that uses a simple rule-based keyword search to streamline pre–magnetic resonance imaging (MRI) safety screening for implantable medical devices, resulting in a 98% reduction in the manual screening workload while maintaining strong performance metrics.

## Introduction

**Figure 1.**
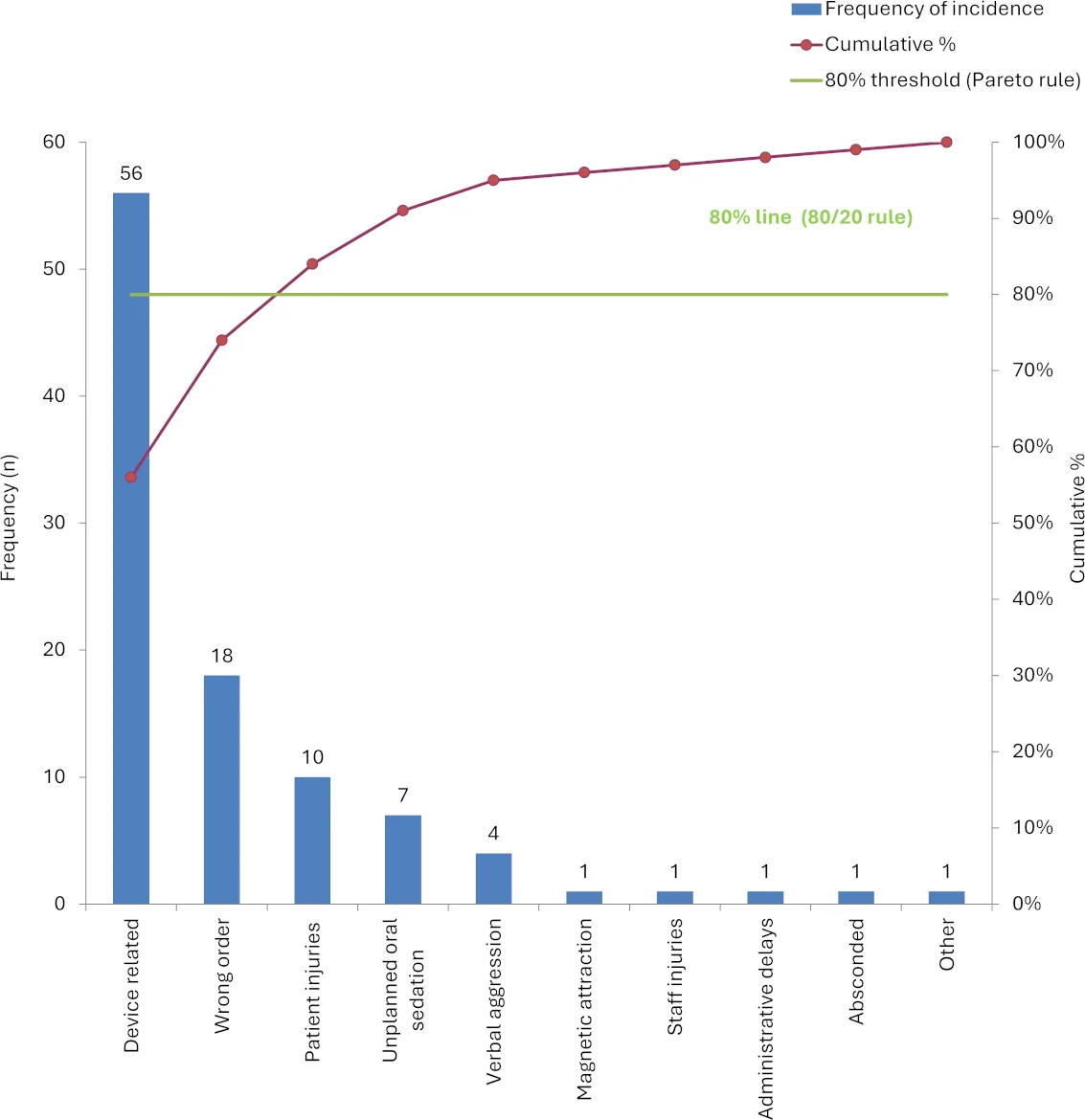
Pareto chart of magnetic resonance imaging (MRI) incident reports (June 2022 to October 2023), showing that 56% of MRI incidents were device related. Green line: 80% threshold (Pareto 80/20 rule). Red line: cumulative percentage of incident reports (%).

The global population is aging rapidly, resulting in an increased prevalence of chronic diseases that often necessitate implantation of devices such as pacemakers [1] and programmable ventriculoperitoneal shunts [2]. A significant magnetic resonance imaging (MRI) safety concern arises from the growing prevalence of implantable devices (Multimedia Appendix 1), which can cause heating, unintended stimulation, alteration of device settings, and malfunction [3-5]. Due to high demand and appointment turnaround, patients are scheduled before screening. To mitigate risks, radiographers perform blanket pre-MRI screening by manually reviewing electronic medical records (EMRs), surgical histories, device records, and prior imaging reports 2 days before the scheduled appointment. However, this is inefficient; radiographers timed during reviews took approximately 400 minutes daily (~2.5 min/patient for 160 patients). Discovering devices on arrival caused delays, last-minute cancellations, or unsafe scans, compromising care and patient experience [6,7]. An institutional Pareto analysis of MRI incidents showed that 56% were device related (Figure 1). Of these, 67.85% were due to undisclosed devices, with pacemakers being the most frequent contributor (73.21%). These on-arrival discoveries resulted in approximately 13 cancellations annually, wasting an estimated 494 minutes of scanner capacity (annualized from the Pareto period).

## Methods

We implemented a dashboard powered by Endeavour AI (National University Health System), a real-time data analytics platform integrating Spotfire (TIBCO Software Inc) streaming analytics. The dashboard consolidates key Epic (Epic Systems Corp) data streams—appointments, device records, orders, and radiology reports—with nightly refresh for data processing (Multimedia Appendix 2).

A rule-based keyword-matching algorithm using regular expressions was applied to detect device-related terms (Multimedia Appendix 3). When a match was detected, the patient was automatically flagged, with device type and radiology report previews for verification (Multimedia Appendix 4).

Performance metrics (sensitivity, specificity, positive predictive value [PPV], and negative predictive value [NPV]) were calculated based on unique visits. Confirmation of devices at the final MRI safety interview was used as the reference standard; for nonattenders, EMR review and prior imaging were used.

Following keyword refinements in June 2024, improvements were compared between Q2 (February-April 2024) and Q4 (August-October 2024) using pooled quarterly metrics. Statistical comparisons were performed by applying 2-proportion Z-tests to quarterly performance metrics. Statistical analyses were performed using Microsoft Excel.

A retrospective audit of device prevalence and disclosure rates was conducted over the same period. A user survey was also administered postdeployment using a 5-point Likert scale to assess usability and workflow integration. The survey was distributed to all 30 MRI radiographers, with 28 responses received (response rate 93.3%). Results are reported as top-box scores (proportion rating ≥4, ie, agree or strongly agree) with median and IQR. We continued tracking false negatives, device-related incidences, and operational consequences for a year postimplementation, from November 2024 to October 2025.

### Ethical Considerations

A formal review was waived by the National Healthcare Group Domain-Specific Review Board, as patients were not subjected to additional risks beyond usual clinical practice (NHG DSRB reference number: 2025‐0522).

## Results

Between November 2023 and October 2024, 32,214 unique patient visits were prospectively evaluated (Multimedia Appendix 5). The dashboard flagged only 1.5% of visits (2‐3 patients/day). Radiology report previews provided direct traceability in the EMR, reducing screening time from 2.5 to 1.5 minutes/patient, cutting daily screening time from 400 minutes to less than 5 minutes (1.5 min/patient × 3 flagged patients = 4.5 min). This represented a 98% reduction in screening workload, corresponding to 0.78 full-time equivalent savings annually. Early identification of 38 undisclosed devices prevented 1280 minutes of scheduled scanner time loss and reduced on-arrival disruptions, enabling proactive appointment substitution with adequate preparation time.

System performance improved following keyword refinement. Comparison between Q2 and Q4 (Multimedia Appendix 6) demonstrated increased PPV (27.93% to 85.71%), improved specificity (98.34% to 99.87%), and reduced false-positive rate (1.66% to 0.13%), all statistically significant (*P*<.05) (Multimedia Appendix 7). These gains reflect an expected trade-off: sensitivity decreased slightly from 92.59% to 90.41%, and NPV decreased marginally from 99.95% to 99.91%; however, neither reduction was statistically significant (*P*=.66 and *P*=.42, respectively), demonstrating that substantial gains in specificity and PPV were achieved without a significant cost to sensitivity or NPV.

False negatives (n=29, 0.09%) were primarily attributed to data and temporal limitations, with 1 multifactorial case involving clinical team nondisclosure at both referral and final safety interview, compounded by a temporal limit (Multimedia Appendix 8). The system’s sensitivity was further demonstrated by its ability to identify 6 devices that were implanted after the MRI was ordered. Final-quarter (Q4) performance reflects the system’s true performance following iterative optimization over the study period (Table 1).

**Table 1.** Dashboard performance metrics over 32,214 unique patient visits.

Performance metric	Measurement
Total flagged by dashboard, n (%)	484 (1.50)
True positives, n (%)	209 (0.65)
False positives, n (%)	275 (0.85)
True negatives, n (%)	31,701 (98.41)
False negatives, n (%)	29 (0.09)
Negative predictive value (%)	
Average across all quarters	99.91
Q4^a^	99.91
Positive predictive value (%)	
Average across all quarters	43.18
Q4^a^	85.71
Sensitivity (%)	
Average across all quarters	87.82
Q4^a^	90.41
Specificity (%)	
Average across all quarters	99.14
Q4^a^	99.87

a Performance metrics for Q4 (August 2024 to October 2024).

The retrospective audit showed that 183/451 (40.58%) devices were undisclosed on request forms (Multimedia Appendix 9). Device prevalence was 1.4%, close to the 1.5% flagged by the dashboard.

Most respondents to the user survey agreed or strongly agreed across all domains (top-box range: 67.9%‐85.7%) (Multimedia Appendix 10). Reduction in manual effort was the highest-rated domain (85.7%, median 5, IQR 4‐5). The dashboard was rated as an important component of daily screening (78.6%, median 4.5, IQR 4‐5). Device type (85.7%) and radiology report preview (82.1%) were highly rated features (both median 4, IQR 4‐5) for screening efficiency. Ease of use (67.9%) and clarity of information (71.4%) had wider IQRs (3‐5), indicating more varied user experiences and identifying areas for future refinement.

Postimplementation tracking recorded 22 false negatives: 15 temporal limits, 6 data gaps, and 1 keyword gap—since corrected (Multimedia Appendix 8). Ten were intercepted in advance through manual review, and 4 were safely accommodated on arrival. There were 13 on-arrival cancellations during the primary study period (680 min of lost scanner time), consistent with the annualized preimplementation baseline (~13 per year, ~494 min). This fell to 8 cancellations (360 min) during the postimplementation tracking year.

## Discussion

This shift from blanket to targeted, data-driven triaging demonstrates that high-volume radiology departments can achieve substantial efficiency gains. The 98% workload reduction is an operational improvement, freeing radiographers for clinical tasks, reducing cognitive overload, and recovering scanner capacity, with direct implications for resource allocation and revenue protection. As MRI demand and device prevalence rise with the aging population, this scalable framework leverages existing EMR infrastructure without using complex AI models, ensuring long-term operational sustainability.

The dashboard achieved a near-perfect NPV in identifying patients without devices. False positives are mitigated through human-in-the-loop review. As the dashboard cannot parse unstructured notes or confirm device removal, a risk of automation bias exists; this is addressed by positioning the dashboard strictly as a first-line triage tool. Critically, the dashboard does not replace existing safety protocols. In accordance with the American College of Radiology’s *Manual on MR Safety* [8], a mandatory in-person safety interview and physical verification by a radiographer remain standard practice, ensuring that patient safety is not compromised.

Postimplementation tracking confirmed a reduction in device-related events, with extended lead time enabling early multidisciplinary coordination and fewer on-arrival disruptions. Notably, on-arrival cancellations during the primary study period remained consistent with the preimplementation baseline, as the dashboard alone did not eliminate same-day scheduling gaps; the subsequent reduction to 8 on-arrival cancellations postimplementation coincided with awareness initiatives. Same-day urgent appointments will continue to bypass the overnight refresh by clinical necessity, representing a residual risk. Future developments should explore real-time, scheduling-triggered alerts to address this gap. Undisclosed devices remain a systemic challenge beyond institutional control, underscoring the role of data analytics in bridging clinical documentation gaps.

Several limitations exist. Single-institution EMR customization limits broader applicability, necessitating multisite validation, and the locally developed end-user survey was not formally validated, limiting generalizability of self-reported measures. Keyword searching lacks the semantic depth of advanced natural language processing. Relying on EMR reviews for nonattenders introduces differential verification bias, potentially undercounting false negatives in this cohort. False negatives represent a residual baseline risk rooted in temporal and data gaps, partially mitigated by manual review of pre-May 2022 records, though external records remain inaccessible. Importantly, all false negatives were safely intercepted without patient harm by either compulsory manual prescreening for same-day appointments or the mandatory final in-person safety interview.

The dashboard evaluated only 238 of 451 total devices, as same-day appointments and paper-based workflows bypassed electronic detection—though safety was maintained through compulsory manual reviews.

The 1280 minutes represents an upper-bound estimate, as canceled slots may be partially recovered through reactive backfilling. Nonetheless, early device interception enables planned substitution, which is operationally superior to reactive last-minute backfilling.

Future developments should focus on integrating natural language processing to improve clinical context interpretation, as well as integrating the system with national device registries to further enhance MRI safety.

## Supplementary material

10.2196/90391 Multimedia Appendix 1 List of devices of interest during pre–magnetic resonance imaging safety screening.

10.2196/90391 Multimedia Appendix 2 Data analysis workflow for the pre–magnetic resonance imaging screening dashboard.

10.2196/90391 Multimedia Appendix 3 List of device keywords used in the Endeavour AI dashboard.

10.2196/90391 Multimedia Appendix 4 Pre–magnetic resonance imaging safety screening dashboard.

10.2196/90391 Multimedia Appendix 5 Monthly dashboard performance metrics across all magnetic resonance imaging visits between November 2023 and October 2024.

10.2196/90391 Multimedia Appendix 6 Pooled performance metrics by quarters.

10.2196/90391 Multimedia Appendix 7 Statistical comparison of Q2 (February 2024 to April 2024) and Q4 (August 2024 to October 2024) performance metrics following keyword refinements in June 2024, particularly for shunt-related terms.

10.2196/90391 Multimedia Appendix 8 Tracking of false negatives, device-related incidences, and operational consequences of study period (November 2023 to October 2024) vs postimplementation period (November 2024 to October 2025).

10.2196/90391 Multimedia Appendix 9 Retrospective review of monthly trends in gross device prevalence and disclosure rates over the 1-year study period.

10.2196/90391 Multimedia Appendix 10 Results for postimplementation survey.

## Abbreviations

EMR: electronic medical record
MRI: magnetic resonance imaging
NPV: negative predictive value
PPV: positive predictive value
