# Supplementary material for: Data Analytics Dashboard for Pre–Magnetic Resonance Imaging Safety Screening of Implantable Medical Devices
Source: J Med Internet Res. 2026 Sep 23;28:e90391. doi: 10.2196/90391 (PMC13600589; doi:10.2196/90391)
Supplement: Supplementary file 1 [file jmir-v28-e90391-s001.docx]

During pre-MRI safety screening, devices are categorized into Active Implantable Medical Devices (AIMDs) and Passive Medical Devices (PMDs) due to the safety risks they pose in the magnetic resonance environment. We have chosen to focus on complex devices while intentionally excluding orthopaedic hardware as complex devices carry significant MRI safety risks and require strict multidisciplinary coordination, consents, dedicated pre and post scan checks or specific scanner allocations.

| **Active Implantable Medical Devices (AIMD)** are devices that rely on an electrical power source, programmability, or telemetry capabilities. These devices demand strict multidisciplinary coordination, consent, including pre- and post-MRI safety checks by vendor representatives and/or cardiology teams, as well as specific scanner allocations to keep scanning conditions within manufacturer limits.  AIMD of interest:   - **Cardiac Devices**: Pacemakers, Cardiac Implantable Electronic Devices (CIED), Implantable Cardioverter-Defibrillators (ICD), Cardiac Resynchronisation Therapy Defibrillators (CRTD), and Cardiac loop recorders - **Neurostimulators**: Devices such as deep brain, vagal nerve, spinal, bone, and bladder stimulators. - **Infusion Pumps**: Including pain, drug, or insulin pumps. - **Cochlear implants** | **Passive Medical Devices (PMD)** are devices that function without an electrical power source but remain magnetically sensitive or require specific programming conditions.  PMD of interest:   - Programmable Ventriculoperitoneal (VP) shunts: These implants require strict pre- and/or post-MRI checks involving the neurosurgery team to ensure the shunt settings have not been altered by the magnetic field, and they must be scheduled in appropriate 1.5T scanners. - Epicardial pacing wires or stimulator wires: These require specific informed consent prior to the MRI and are restricted to 1.5T scanners. |
| --- | --- |
