# Supplementary material for: Data Analytics Dashboard for Pre–Magnetic Resonance Imaging Safety Screening of Implantable Medical Devices
Source: J Med Internet Res. 2026 Sep 23;28:e90391. doi: 10.2196/90391 (PMC13600589; doi:10.2196/90391)
Supplement: Supplementary file 2 [file jmir-v28-e90391-s002.docx]

The diagram shows how EPIC (Epic Systems Corporation, Verona, Wisconsin, USA) data streams to TIBCO Spotfire (TIBCO Software Inc., Palo Alto, California, USA) and Endeavour AI (National University Health System, Singapore) in an overnight data refresh. An automated keyword search using a predefined list of device flags at-risk patients for radiographer review on the dashboard. The EAI dashboard provides an overview of patients scheduled from 1 to 14 days ahead. Importantly, the dashboard does not replace MR safety protocols. In accordance with the ACR Manual on MR Safety, a mandatory final in person safety interview and physical verification by a radiographer immediately before entering Zone 4 remains standard practice, ensuring patient safety is not compromised.
