# Supplementary material for: Data Analytics Dashboard for Pre–Magnetic Resonance Imaging Safety Screening of Implantable Medical Devices
Source: J Med Internet Res. 2026 Sep 23;28:e90391. doi: 10.2196/90391 (PMC13600589; doi:10.2196/90391)
Supplement: Supplementary file 3 [file jmir-v28-e90391-s003.docx]

| Active Implantable Medical Devices**:** - Pacemaker, ppm, cied, aicd, single lead, single-lead, double lead, double-lead, triple lead, triple-lead, crt-d, crtd - Stimulator - Cochlear implant, otologic - Pump | Passive Medical Devices: - VP shunt, shunt tub^a^, shunt tip, ventriculo-peritoneal, ventriculoperitoneal, ventriculo peritoneal, programmable - Pacing wire |
| --- | --- |

^a^ 'Shunt tub' was intentionally deployed as a substring to capture all variants including 'shunt tube' and 'shunt tubing'.
