# Supplementary material for: Data Analytics Dashboard for Pre–Magnetic Resonance Imaging Safety Screening of Implantable Medical Devices
Source: J Med Internet Res. 2026 Sep 23;28:e90391. doi: 10.2196/90391 (PMC13600589; doi:10.2196/90391)
Supplement: Supplementary file 4 [file jmir-v28-e90391-s004.docx]

When a keyword match is detected, the patient is automatically flagged on the dashboard, displaying appointment details, device type, and a preview of the related radiology report for pre-MRI safety review by the radiographer.
