# Supplementary material for: Data Analytics Dashboard for Pre–Magnetic Resonance Imaging Safety Screening of Implantable Medical Devices
Source: J Med Internet Res. 2026 Sep 23;28:e90391. doi: 10.2196/90391 (PMC13600589; doi:10.2196/90391)
Supplement: Supplementary file 5 [file jmir-v28-e90391-s005.docx]

Device detection outcomes across 32,214 unique patient visits, comprising true positives (n=209), false positives (n=275), false negatives (n=29), and true negatives (n=31,701).

|  | Nov-23 | Dec-23 | Jan-24 | Feb-24 | Mar-24 | Apr-24 | May-24 | Jun-24 | Jul-24 | Aug-24 | Sept-24 | Oct-24 | Total |
| --- | --- | --- | --- | --- | --- | --- | --- | --- | --- | --- | --- | --- | --- |
| Unique visits (N) * | 2613 | 2436 | 2859 | 2515 | 2618 | 2703 | 2721 | 2502 | 2980 | 2836 | 2682 | 2749 | 32,214 |
| EAI flagged | 38 | 58 | 55 | 65 | 64 | 50 | 43 | 15 | 19 | 18 | 18 | 41 | 484 (1.5%) |
| EAI missed (FN) | 6 | 2 | 4 | 4 | 0 | 0 | 3 | 1 | 2 | 1 | 4 | 2 | 29 (0.09%) |
| TP | 15 | 18 | 14 | 17 | 18 | 15 | 17 | 12 | 17 | 16 | 15 | 35 | 209 (0.65%) |
| FP | 23 | 40 | 41 | 48 | 46 | 35 | 26 | 3 | 2 | 2 | 3 | 6 | 275 (0.85%) |
| TN | 2569 | 2376 | 2800 | 2446 | 2554 | 2653 | 2675 | 2486 | 2959 | 2817 | 2660 | 2706 | 31,701 (98.41%) |
| EAI devices cancelled | 6 | 1 | 3 | 4 | 2 | 0 | 2 | 3 | 4 | 1 | 4 | 8 | 38 |
| Time saved (mins)# | 180 | 40 | 70 | 220 | 40 | 0 | 60 | 110 | 150 | 30 | 150 | 230 | 1280 mins |
| Cancelled on arrival | 3 | 2 | 1 | 2 | 0 | 0 | 2 | 0 | 2 | 1 | 0 | 0 | 13 |
| NPV | 99.77% | 99.92% | 99.86% | 99.84% | 100.00% | 100.00% | 99.89% | 99.96% | 99.93% | 99.96% | 99.85% | 99.93% | Average: 99.91% Q4: 99.91%^ |
| PPV | 39.47% | 31.03% | 25.45% | 26.15% | 28.13% | 30.00% | 39.53% | 80.00% | 89.47% | 88.89% | 83.33% | 85.37% | Average: 43.18% Q4: 85.71%^ |
| Sensitivity | 71.43% | 90.00% | 77.78% | 80.95% | 100.00% | 100.00% | 85.00% | 92.31% | 89.47% | 94.12% | 78.95% | 94.59% | Average: 87.82% Q4: 90.41%^ |
| Specificity | 99.11% | 98.34% | 98.56% | 98.08% | 98.23% | 98.70% | 99.04% | 99.88% | 99.93% | 99.93% | 99.89% | 99.78% | Average: 99.14% Q4: 99.87%^ |

* Performance metrics was based on unique visits to avoid inflating workload when multiple scans occurred within a single encounter. The final in-person safety interview was used as the reference standard; for non-attenders, EMR review and prior imaging. # Time saved calculated by summing institutional procedure time by region (e.g., 30 mins for Brain, 50 mins for Prostate). ^Performance metrics for Q4 (August 2024 to October 2024). Q1: November 2023 to January 2024, Q2: February 2024 to April 2024, Q3: May 2024 to July 2024, Q4: August 2024 to October 2024.
