# Supplementary material for: Data Analytics Dashboard for Pre–Magnetic Resonance Imaging Safety Screening of Implantable Medical Devices
Source: J Med Internet Res. 2026 Sep 23;28:e90391. doi: 10.2196/90391 (PMC13600589; doi:10.2196/90391)
Supplement: Supplementary file 6 [file jmir-v28-e90391-s006.docx]

The 95% confidence intervals were calculated using the exact binomial method, calculated with Excel BETAINV formulas. Performance metrics were calculated based on the last quarter (August 24 to October 24). Q1: November 2023 to January 2024, Q2: February 2024 to April 2024, Q3: May 2024 to July 2024, Q4: August 2024 to October 2024.

|  | **Sensitivity** | **Specificity** | **PPV** | **NPV** |
| --- | --- | --- | --- | --- |
| Q1: Nov-23, Dec-23, Jan-24 | 79.66%  **Lower CI:** 67.17% **Upper CI:** 89.02% | 98.67%  **Lower CI:** 98.40%  **Upper CI:** 98.92% | 31.13%  **Lower CI:** 23.85%  **Upper CI:** 39.16% | 99.85%  **Lower CI:** 99.73%  **Upper CI:** 99.92% |
| Q2: Feb-24, Mar-24, Apr-24 | 92.59%  **Lower CI:** 82.11% **Upper CI:** 97.94% | 98.34%  **Lower CI:** 98.03%  **Upper CI:** 98.61% | 27.93%  **Lower CI:** 21.50%  **Upper CI:** 35.12% | 99.95%  **Lower CI:** 99.87%  **Upper CI:** 99.99% |
| Q3: May-24, Jun-24, Jul-24 | 88.46%  **Lower CI:** 76.56% **Upper CI:** 95.65% | 99.62%  **Lower CI:** 99.46%  **Upper CI:** 99.74% | 59.74%  **Lower CI:** 47.94%  **Upper CI:** 70.77% | 99.93%  **Lower CI:** 99.84%  **Upper CI:** 99.97% |
| Q4: Aug-24, Sept-24, Oct-24 | 90.41% ^  **Lower CI:** 81.24% **Upper CI:** 96.06% | 99.87% ^  **Lower CI: 99.76%**  **Upper CI:** 99.93% | 85.71% ^  **Lower CI:** 75.87%  **Upper CI:** 92.65% | 99.91% ^  **Lower CI:** 99.82%  **Upper CI:** 99.97% |
