# Supplementary material for: Data Analytics Dashboard for Pre–Magnetic Resonance Imaging Safety Screening of Implantable Medical Devices
Source: J Med Internet Res. 2026 Sep 23;28:e90391. doi: 10.2196/90391 (PMC13600589; doi:10.2196/90391)
Supplement: Supplementary file 7 [file jmir-v28-e90391-s007.docx]

| **Metric** | **Q2 Performance** | **Q4 Performance** | **Z statistic** | **Two-sided P-value** |
| --- | --- | --- | --- | --- |
| **Specificity** | 98.34% | 99.87% | -10.137 | *P*<.001 |
| **PPV** | 27.93% | 85.71% | -11.090 | *P*<.001 |
| **FPR** | 1.66% | 0.13% | 10.137 | *P*<.001 |
| **NPV** | 99.95% | 99.91% | 0.800 | *P*=.42 |
| **Sensitivity** | 92.59% | 90.41% | 0.440 | *P*=.66 |
