# Supplementary material for: Data Analytics Dashboard for Pre–Magnetic Resonance Imaging Safety Screening of Implantable Medical Devices
Source: J Med Internet Res. 2026 Sep 23;28:e90391. doi: 10.2196/90391 (PMC13600589; doi:10.2196/90391)
Supplement: Supplementary file 8 [file jmir-v28-e90391-s008.docx]

**Part A:** False Negative Root Cause Analysis

| **Root Cause** | **Study Period n=29** | **Post-Implementation n=22** |
| --- | --- | --- |
| **Temporal limits**: Urgent, same-day appointments (often inpatients) that bypassed the overnight electronic data refresh. | n=8 (27.6%) | n=15 (68.2%) |
| **Data limits**: Missing or inaccessible data streams, including legacy records predating EMR adoption in May 2022, devices documented only in scanned PDFs, and records from external institutions. | n=20 (69.0%) | n=6 (27.3%) |
| **Multifactorial**: non-disclosure by clinical team at referral and final safety interview, compounded by temporal limit ^a^. | n=1(3.4%) | - |
| **Keyword gap** (post-implementation only): A device documented as "nerve stimulation implantation device" was not captured by the existing 'stimulator' keyword, which has been updated | — | n=1 (4.5%) |

^a^ A paediatric intubated ICU patient underwent VP shunt revision from non-programmable to programmable the previous day; the device change was not declared on the MRI request form, and the same-day MRI order bypassed the overnight data refresh. During the final safety interview, the accompanying clinical team, who bear primary responsibility for declaring device status for patients under their care, did not disclose the revision; they only recalled the revision after scout image artefacts were identified. The patient was immediately removed from the MRI suite without harm.

**Part B:** Operational Consequences of False Negatives

| **Operational Consequences** | **Study Period n=29** | **Post-Implementation n=22** |
| --- | --- | --- |
| **Intercepted in advance:** Devices identified by radiographers during manual review and subsequently managed or cancelled well ahead of the patient's arrival | n=13 (44.8%) | n=10 (45.5%) |
| **On-arrival accommodations:** Same-day multidisciplinary coordination and informed consent arranged on-arrival for:   - Study period: 2 neurostimulators, 1 programmable VP shunt - Post-implementation: 1 programmable VP shunt, 1 cochlear implant, 1 pacing wire, 1 hypoglossal nerve stimulator | n=3 (10.3%) | n=4 (18.2%) |
| **On-arrival cancellations:** Unmitigable cancellations involving undisclosed devices that could not be safely accommodated on the spot | n=13 (44.8%) | n=8 (36.4%) |
| **Scanner time lost due to on-arrival cancellations** | 680 mins | 360 mins |
