# Supplementary material for: Data Analytics Dashboard for Pre–Magnetic Resonance Imaging Safety Screening of Implantable Medical Devices
Source: J Med Internet Res. 2026 Sep 23;28:e90391. doi: 10.2196/90391 (PMC13600589; doi:10.2196/90391)
Supplement: Supplementary file 9 [file jmir-v28-e90391-s009.docx]

Gross device includes patients with devices that were given manual appointments using hardcopy forms that are not captured digitally in EPIC data and same day add-on patients who missed the overnight data refresh. An average of 40.58% of devices remain undisclosed at the time of ordering. The average prevalence of devices in our patient population was 1.4%.

|  | Nov-23 | Dec-23 | Jan-24 | Feb-24 | Mar-24 | Apr-24 | May-24 | Jun-24 | Jul-24 | Aug-24 | Sept-24 | Oct-24 | Total |
| --- | --- | --- | --- | --- | --- | --- | --- | --- | --- | --- | --- | --- | --- |
| Number of devices | 40 | 46 | 40 | 31 | 39 | 35 | 35 | 25 | 39 | 35 | 38 | 48 | 451^a^ |
| Number of disclosed devices | 21 | 32 | 26 | 18 | 21 | 26 | 19 | 11 | 26 | 21 | 18 | 29 | 268 |
| Number of undisclosed devices | 19 | 14 | 14 | 13 | 18 | 9 | 16 | 14 | 13 | 14 | 20 | 19 | 183 |
| Undisclosed devices (%) | 47.50 | 30.43 | 35.00 | 41.94 | 46.15 | 25.71 | 45.71 | 56.00 | 33.33 | 40.00 | 52.63 | 39.58 | **Average: 40.58%** |
| Prevalence of devices (%) | 1.53 | 1.89 | 1.40 | 1.23 | 1.49 | 1.29 | 1.29 | 1.00 | 1.31 | 1.23 | 1.42 | 1.75 | **Average: 1.40%** |

ᵃ The gross device total (n=451) includes all devices identified during the retrospective audit, comprising both electronically evaluated patients and those processed via hardcopy forms (e.g., manual CIED referrals) or same day add-on appointments that bypassed the overnight electronic data refresh. The dashboard evaluated only 238 of these devices (209 true positives and 29 false negatives), as the remainder bypassed electronic detection entirely. Devices processed via paper-based workflows are inherently disclosed and managed through compulsory manual review and therefore do not represent a patient safety gap.
