# Supplementary material for: Data Analytics Dashboard for Pre–Magnetic Resonance Imaging Safety Screening of Implantable Medical Devices
Source: J Med Internet Res. 2026 Sep 23;28:e90391. doi: 10.2196/90391 (PMC13600589; doi:10.2196/90391)
Supplement: Supplementary file 10 [file jmir-v28-e90391-s010.docx]

An end-user survey was distributed electronically (via FormSG) four months after the full dashboard deployment. The survey was distributed to all 30 MRI radiographers; 28 responded (response rate 93.3%). The survey employed a 5-point Likert scale (1 = Strongly Disagree, 5 = Strongly Agree). Results are reported as top-box scores (proportion of respondents rating ≥4, i.e., Agree or Strongly Agree) alongside median scores and interquartile ranges (IQR).

Overall, most respondents agreed or strongly agreed across all domains (top-box range: 67.9%–85.7%). 85.7% of respondents agreed that the dashboard reduced manual efforts (Median 5, IQR 4–5). 78.6% of respondents also agreed that the dashboard is an important component of daily screening (Median 4.5, IQR 4–5), reflecting how deeply embedded it has become. Two features of the dashboard, device type (85.7%) and radiology report preview (82.1%) were rated highly (both Median 4.0, IQR 4–5) for supporting screening efficiency and focusing their review within EMR. Ease of use and clarity had wider IQRs (3.0–5.0), suggesting more varied experiences — worth acknowledging as areas for refinement.

Top-box Score (%)

|  | Top-box (≥4) % |
| --- | --- |
| Ease of use | 67.9% |
| Clarity of information | 71.4% |
| Workflow integration | 82.1% |
| Reduction in manual effort | 85.7% |
| Time savings | 78.6% |
| Reliance on dashboard | 72.4% |
| Importance in workflow | 78.6% |
| Device type column | 85.7% |
| Report column | 82.1% |
